# O Parent, Where Art Thou?

**DOI:** 10.1002/pne2.12010

**Published:** 2019-12-20

**Authors:** Alexandra Ullsten, Mats Eriksson, Anna Axelin

**Affiliations:** ^1^ Department of Musicology Örebro University Örebro Sweden; ^2^ Centre for Clinical Research Karlstad Sweden; ^3^ Faculty of Medicine and Health Örebro University Örebro Sweden; ^4^ Department of Nursing Science University of Turku Turku Finland

Neonatal pain researchers in British Columbia, Canada, have designed a “robot” to help babies delivered preterm to cope with painful procedures by mimicking skin‐to‐skin contact with a parent.^1^ A picture of this device with an infant in prone position resting on this appliance was displayed in one of the many cutting‐edge lectures at the excellent and well‐organized 12th International Symposium on Pediatric Pain in Basel in June. This “substitute parent‐device,” shaped like a rectangular platform, fits inside an incubator and is programmed with information on the parent's heartbeat and breathing motions, simulating skin‐to‐skin contact with a parent who may not be available during around‐the‐clock procedures in a neonatal intensive care unit.

In Florida, USA, to assist preterm born infants requiring care in the neonatal intensive care unit (NICU) to eat more efficiently and increase weight gain, music medicine researchers have invented a device to enhance suck effectiveness.^2^ The device is a pacifier that can detect whether a baby is sucking on it, and in turn, the baby gets to hear a lullaby as an auditory input in direct response to effective sucking. The music is prerecorded and consists of instrumental lullabies or songs sung by the parent.

Devices such as the ones identified have good intentions and promising results in research studies.^1^, ^2^, ^3^ These substitutes are also said to save millions of dollars in staffing costs and shorten hospital stays.^4^ However, devices like these fail to acknowledge the needs of the whole family. Given the high incidence of maternal depression, family stress, and elevated incidence of post‐traumatic stress in NICU parents, the benefits and cost‐effectiveness derived with active parental participation in care are overlooked with these approaches.^5^, ^6^

The hospitalized infant has an innate need for experiencing contingent and reciprocal interactions with a loving and affectionate parent. Similarly, parents have a need to fulfill their protective role. Separation and ruptures in the processes and functions linked to attachment, for example, related to painful procedures, may have long‐term negative effects.^7^ The researchers in Canada emphasize in their study that parents should always be the first choice.^1^ Why then, develop devices that we know will deprive the infant of optimal and efficacious pain alleviation and development,^5^ including threatening parents’ mental well‐being? What if the hospital boards find these devices more convenient and less expensive in the neonatal care than trying to change societal structures in the healthcare system and rebuild hospitals to welcome parents around the clock? More and more research emphasizes the importance of parents as mediators for pain relief.^8^, ^9^, ^10^, ^11^ Parents are an underused resource in pain management, but they are highly motivated to participate in their infant's pain care.^12^

Pain management should be considered a reciprocal continuation where both parent and infant can learn from and help each other. Touch is the sense that develops first and provides important means for interaction.^13^ It mediates the feeling of security to the infant through the activation of the parasympathetic nervous system^14^ and alleviates pain, for example, through oxytocinergic mechanisms.^15^ Facilitated tucking^16^ and skin‐to‐skin care^17^ are effective pain management methods that give parents an opportunity to protect their infant from harm while alleviating their own anxiety^18^ and developing their parenting skills,^12^ unlike utilizing devices that only mimic parent's touch.

Infants are multisensory, biopsychosocial beings. Parent‐driven nonpharmacological pain‐alleviating interventions, including the parent's voice, engage all senses at the same time facilitating pain relief.^19^ A parent's voice, along with facial and gestural expressions, is communicative in a multisensory and multimodal manner. The first voice the fetus hears and ascribes significance to is the mother's voice and the music of her prosody.^20^ The musical qualities of the parent's voice are salient in the perinatal experience of speech, enculturation, attachment, and safety. In music medicine research, recorded music and recorded mother's voice during skin puncture are considered simple, convenient, inexpensive, and complication‐free interventions.^21^, ^22^, ^23^ Recordings are convenient for the researcher or the NICU staff, but do not acknowledge the needs of the family‐infant unit. Infant‐directed communication, both speech and singing, can be simulated in recordings but are under no circumstances multisensory, interactive, or responsive to an infant in pain and cannot share the infant's pain experience.^19^

The public healthcare system with family‐friendly parental leave policies might be one of the reasons that the Nordic countries today are at the forefront of welcoming and including parents and partners in the care of their infant around the clock.^6^, ^24^, ^25^, ^26^ Even if most infants are still cared for in traditional multi‐bed, open‐bay NICUs, the awareness of family‐centered care is very high and in constant growth in Denmark, Finland, Iceland, Norway, and Sweden. Parents are welcome to stay close to their infant most of the time with no restrictions in visiting hours. More and more NICUs in the Nordic hospitals are today built (or rebuilt) to welcome parents around the clock, with separate family rooms, couplet care for mother and infant with zero separation, bedside rounds, and opportunities for siblings to stay in family rooms with no restrictions on visiting hours.^24^ In line with the Nordic approach, the current best evidence trend in the NICU care units in North America is the family integrated care philosophy, emphasizing the well‐being of the family‐infant unit.^27^

What seems to be most crucial for an infant before, during, and after a painful situation as well as for future painful experiences is the extent to which the parent is emotionally available and stable, capable of noticing and contingently interpreting cues in the infant's behavior, responding adequately to the infant's distress signals and able to soothe, regulate, and share the infant's states.^28^ For various reasons, not all parents are able to provide emotional and physical closeness, not even in the context of the Nordic public health care. In these cases, are substitute parent‐devices the only solution? Or, perhaps we could consult the guidelines,^29^ which for example recommend including strength‐based psychoeducation service for parents and family support programs in clinical care.

Imagine if neonatal researchers used their time and research funding to translate research into practice promoting infant‐parent closeness during painful procedures instead of researching devices that will replace parents with unresponsive robots and music machines. Hospitalized infants need their parents to be present, especially in painful situations, and the parents need to take care of their infant and protect the infant from pain and stress. The scientific rationales for involving parents in all neonatal care and specifically in neonatal pain management are already verified. Parents are the primary source for pain alleviation, nutrition, protection, love, and development, and every possible action should be made to promote this!

## CONFLICTS OF INTEREST

The authors have no conflicts of interest to declare.
